# Comparison of Anti-Inflammatory and Antibacterial Properties of *Raphanus sativus* L. Leaf and Root Kombucha-Fermented Extracts

**DOI:** 10.3390/ijms25115622

**Published:** 2024-05-22

**Authors:** Aleksandra Ziemlewska, Martyna Zagórska-Dziok, Agnieszka Mokrzyńska, Zofia Nizioł-Łukaszewska, Dariusz Szczepanek, Ireneusz Sowa, Magdalena Wójciak

**Affiliations:** 1 Department of Technology of Cosmetic and Pharmaceutical Products, Medical College, University of Information Technology and Management in Rzeszow, Sucharskiego 2, 35-225 Rzeszow, Poland; aziemlewska@wsiz.edu.pl (A.Z.); mzagorska@wsiz.edu.pl (M.Z.-D.); amokrzynska@wsiz.edu.pl (A.M.); zniziol@wsiz.edu.pl (Z.N.-Ł.); 2 Department of Neurosurgery and Paediatric Neurosurgery, Medical University of Lublin, 20-090 Lublin, Poland; dariusz.szczepanek@umlub.pl; 3 Department of Analytical Chemistry, Medical University of Lublin, Aleje Raclawickie 1, 20-059 Lublin, Poland; i.sowa@umlub.pl

**Keywords:** *Raphanus sativus* L., radish, kombucha, fibroblasts, keratinocytes, antioxidants, cytotoxicity, pro-inflammatory cytokines, antibacterial activity, skin hydration

## Abstract

In the cosmetics industry, the extract from *Raphanus sativus* L. is fermented using specific starter cultures. These cosmetic ingredients act as preservatives and skin conditioners. Kombucha is traditionally made by fermenting sweetened tea using symbiotic cultures of bacteria and yeast and is used in cosmetic products. The aim of this study was to evaluate the cosmetic properties of radish leaf and root extract fermented with the SCOBY. Both unfermented water extracts and extracts after 7, 14, and 21 days of fermentation were evaluated. The analysis of secondary plant metabolites by UPLC-MS showed higher values for ferments than for extracts. A similar relationship was noted when examining the antioxidant properties using DPPH and ABTS radicals and the protective effect against H_2_O_2_-induced oxidative stress in fibroblasts and keratinocytes using the fluorogenic dye H_2_DCFDA. The results also showed no cytotoxicity to skin cells using Alamar Blue and Neutral Red tests. The ability of the samples to inhibit IL-1β and COX-2 activity in LPS-treated fibroblasts was also demonstrated using ELISA assays. The influence of extracts and ferments on bacterial strains involved in inflammatory processes of skin diseases was also assessed. Additionally, application tests were carried out, which showed a positive effect of extracts and ferments on TEWL and skin hydration using a TEWAmeter and corneometer probe. The results obtained depended on the concentration used and the fermentation time.

## 1. Introduction

Botanical ingredients are a primary source of materials used in both the cosmetic and pharmaceutical sectors. In recent years, there has been an increased interest in plant products known for their skin care properties. These plant-derived materials are used not only in topical skin care but also to help treat various skin ailments. Their significant advantage is their gentle but effective nature, offering safety and non-toxicity without negative side effects. Cosmetics products enriched with bioactive compounds adapt well to the skin’s requirements and are more environmentally sustainable compared with conventional alternatives. Among the widely used natural cosmetic ingredients are plant extracts containing a multitude of bioactive substances that have a significant effect on human skin. These extracts exhibit a variety of properties that include therapeutic applications for specific inflammatory skin diseases such as acne, psoriasis, or atopic dermatitis, as well as skin care functions such as antioxidant, antimicrobial, regenerating, moisturizing, smoothing, cleansing, and brightening effects [1,2,3]. One of the methods used to obtain biologically active compounds is fermentation, which can improve the quality of the product and facilitate the absorption of active substances by the human body. Fermented plants are an innovative trend in cosmetics that is gaining popularity due to their exceptional skin care properties. They are the result of a natural fermentation process in which plant ingredients are biodegraded by microorganisms (bacteria or yeast), most often from the genus *Lactobacillus* spp., *Bifidobacterium* spp., and *Saccharomyces cerevisiae*, as well as the kombucha tea fungus (SCOBY) [4,5]. 

Traditionally, kombucha is prepared using Chinese tea (*Camellia sinensis*) and sugar, which are used as substrates by the SCOBY. Fermentation takes place for 7–20 days at room temperature (18–26 °C). The acidic reaction and strong symbiosis between the bacteria and the yeast in the culture medium prevent the growth of pathogenic microorganisms [6,7]. As a result, a drink is obtained that is rich in acetic bacteria, mainly from the genus *Acetobacter* and *Gluconobacter*, lactic acid bacteria, and yeast, as well as organic acids such as acetic, gluconic, and glucuronic acids, polyphenolic compounds, minerals (iron, copper, manganese, nickel, and zinc), or vitamins C, B1, B2, B6, and B12 [8,9,10]. Thanks to the presence of nutrients, especially organic acids and sugar, a refreshing, slightly sweet and sour drink is created. In this context, kombucha has become one of the promising fermentation products due to its beneficial antioxidant, antibacterial, anti-inflammatory, and hepatoprotective properties [11,12,13]. Currently, alternative plant raw materials that can be subjected to fermentation are being sought. The most frequently mentioned materials in the literature include black and green tea, Jerusalem artichoke and grape extract, milk, red and white wine, Coca-Cola, and fresh sweet whey.

Taking into account the possibility of using substrates other than tea for fermentation, the possibility of using radish leaf and root extract as a fermentation substrate was assessed. Radish (*Raphanus sativus* L.) is a root vegetable belonging to the *Brassicaceae* family and is cultivated and consumed worldwide, although it is not common in some populations. Additionally, the plant contains various vitamins (B1, B2, B3, B5, B6, B9, and C) and minerals (calcium, iron, magnesium, manganese, zinc, potassium, and phosphorus) [14]. The main bioactive compounds are glucosinolates and polyphenols, including phenolic acids and flavonoids, mainly in the form of kaempferol, as well as catechin [15,16]. In addition to the roots, leaves and shoots are also nutritional and medicinally significant [14]. Radish extracts have a historical usage in treating various ailments including stomach diseases, constipation, urinary tract infections, hepatitis, heart disease, and ulcers. Furthermore, several studies have highlighted the antibacterial, anticancer, antioxidant, and anxiety-reducing properties of radish [17,18,19,20,21].

Extracts from *R. sativus* L. are known for their beneficial properties and are used in the cosmetic industry as preservatives, antioxidants, and skin conditioners. In particular, they are known as cosmetic raw materials fermented with bacterial strains of the genus *Lactobacillus* and *Leuconostoc* [22]. In addition, they are subjected to fermentation with specific starter cultures. The Cosmetic Ingredient Review organization assessed the safety of seven ingredients derived from radish roots, all of which were from the species *R. sativus* L. The analysis concluded that the available data were sufficient to assess the safety of all seven ingredients, most of which are considered hair and skin conditioners in cosmetic preparations. Specifically, *Lactobacillus/Radish Root Extract Filtrate* is identified as an antimicrobial agent, while *Leuconostoc/Radish Root Extract Filtrate* acts as both an anti-dandruff and an antifungal agent. Additionally, *Lactobacillus/Radish Root Ferment Filtrate* is exclusively labeled as a preservative, and *R. sativus* L. root extract is also recognized for its antioxidant properties [23,24,25,26,27,28].

The aim of our study was to evaluate the biological properties of fermented radish leaf and root extracts using the kombucha tea fungus for use in cosmetic products. The fermentation process was carried out for periods of 7, 14, and 21 days. The research determined the content of biologically active compounds, antioxidant properties, and the level of reactive oxygen species in skin cells exposed to the tested extracts and ferments. For the potential use of the fermented extract in cosmetic products, cytotoxicity against normal skin cell lines was tested. Moreover, the tested samples were analyzed for the ability to inhibit the activity of cytokines (interleukin-1β and cyclooxygenase-2) in LPS-treated fibroblasts involved in the inflammatory process. Microbiological tests were also carried out using bacterial strains involved in the inflammatory processes of skin diseases. Additionally, the influence of the obtained ferments on hydration and transepidermal water loss was assessed.

## 2. Results and Discussion

### 2.1. Determination of Bioactive Compounds

Chromatographic separation of the components of extracts from the leaves and roots of *R. sativus*, as well as fermented extracts, was conducted using the RP-UPLC MS method. Identification was based on spectrophotometric spectra and mass spectrometry data and comparison with standards or literature reports [14,29]. The results are summarized in Table 1 and Table 2.

The extract from radish leaves mainly contained caffeoylmalic acid; p-coumaroylmalic acid; feruloylmalic acid; rutoside; derivatives of kaempferol, with their contents ranging from approximately 4 to 6.5 µg/g; and kaempferitrin, which was found to be the dominant compound with a content above 9.5 µg/g. Chromatographic profiles obtained for the ferments were richer, additionally confirming the presence of catechins, epicatechins, and gallocatechins, which proved to be the main metabolite (content approximately 24 µg/g). However, the fermentation time had only a slight impact on its content. Additionally, fatty acids (above 55 min) were present in both extracts and ferments.

Generally, the chromatographic profile of the root sample was poorer compared with the profile obtained for the leaf extract, both qualitatively and quantitatively (Figures S1 and S2). Especially for samples not subjected to fermentation, only a few signals confirming the presence of gallocatechins and fatty acids were obtained. Additionally, chromatograms obtained for the ferments showed the presence of, among others, gallocatechin, catechin, epicatechin, rutin, quercetin glucoside, and gallic acid, and their contents generally increased with the fermentation time. In the root samples of radish, the presence of several compounds (at retention times from 30 to 43 min) with absorption maxima in the visible light in the range of 500–515 nm (Figure S3) was observed. These compounds belonged to high-molecular-weight anthocyanin derivatives of pelargonidin with a characteristic mass fragment at *m*/*z* +H 271.05971 (estimated formula: C_15_H_11_O_5_+, error—−1.44 ppm). The amount of these compounds did not increase with fermentation time.

### 2.2. Assessment of Antioxidant Activity

#### 2.2.1. ABTS and DPPH Radical Scavenging

To assess the antioxidant activity of extracts and ferments from the leaves and roots of *R. sativus* L., the ABTS and DPPH methods were used. The ABTS assay is a spectrophotometric method of determining antioxidant capacity to neutralize the blue cation radical generated from ABTS under the influence of sodium persulfate, characterized by a decrease in solution absorbance. The DPPH assay is a spectrophotometric method of determining antioxidant capacity to neutralize the purple DPPH radical. In this method, antioxidants change the color of the solution from purple to yellow.

**Figure 1:**
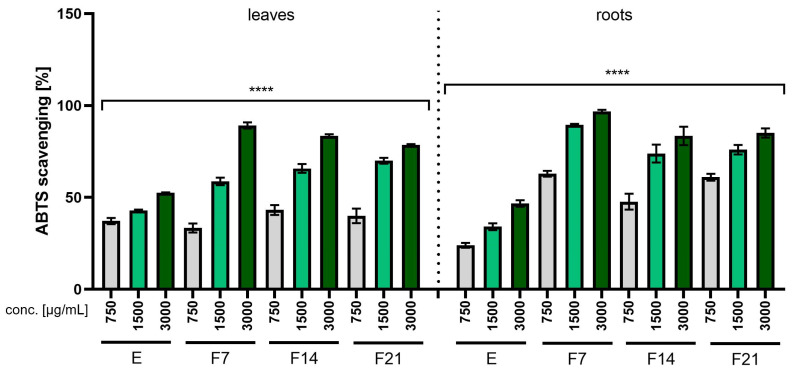
The ability to scavenge the ABTS free radical by *R. sativus* L. leaf and root extracts (E) and ferments (F7, F14, and F21) at the concentration of 750, 1500, and 3000 µg/mL. Data are presented as mean ± SD from three independent experiments, with each sample tested in triplicate. **** *p* < 0.0001.

**Figure 2:**
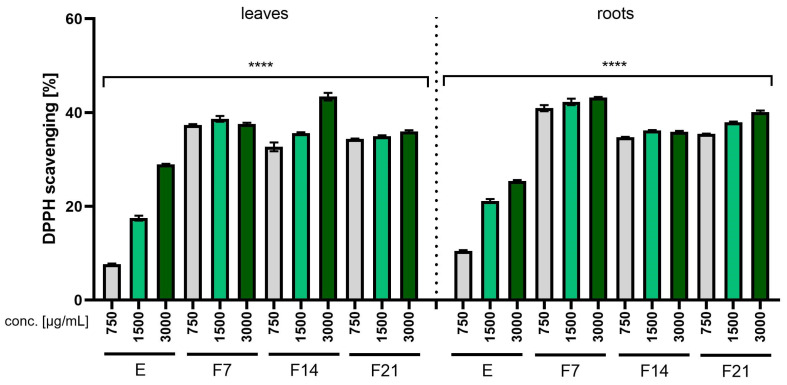
The ability to scavenge the DPPH free radical by *R. sativus* L. leaf and root extracts (E) and ferments (F7, F14, and F21) at the concentration of 750, 1500, and 3000 µg/mL. Data are presented as mean ± SD from three independent experiments, with each sample tested in triplicate. **** *p* < 0.0001.

The antioxidant properties of the *R. sativus* L. extracts and ferments were analyzed at the concentrations 750, 1500, and 3000 µg/mL, and the results are presented as the percentage of free radical scavenging (Figure 1 and Figure 2). The results obtained using the ABTS and DPPH methods significantly indicated that both extracts and ferments had antioxidant properties. In both methods, ferments had stronger antioxidant properties compared with extracts. As the concentration of the extract or ferment increased, an increase in free radical scavenging could be observed. In both methods (ABTS and DPPH), F7 had the strongest antioxidant properties, obtaining 96.8 ± 0.5 and 43.23 ± 0.12%, respectively, for the root at a concentration of 3000 µg /mL.

The results obtained when assessing the antioxidant properties using the ABTS and DPPH methods correspond to the results obtained in the chromatographic analysis (Table 1 and Table 2). As indicated by the literature data and chromatographic analyses, radish extracts and ferments are characterized by the presence of compounds such as gallic acid, catechin, gallocatechin, rutoside, quercetin glucoside, and caffeoylmalic acid, which have proven antioxidant properties [30,31,32]. Kombucha ferments showed higher antioxidant activity compared with non-fermented extracts due to the decomposition of polyphenolic compounds into smaller phenolic substances by microbial enzymes present in the biofilm formed by bacteria and yeast during fermentation. As a result, this process resulted in an overall increase in the total amount of phenols (Table 1 and Table 2) [13,33].

#### 2.2.2. Intracellular ROS Levels in Skin Cells

Oxidative stress caused by excessive amounts of reactive oxygen species affects cells, causing oxidative damage to lipids, proteins, and DNA, which ultimately leads to cell and tissue damage [34]. An imbalance between the amount of free radicals and the concentration of antioxidants adversely affects the condition and functioning of the skin, and oxidative stress is one of the main causes of skin aging [35]. It has been proven that the antioxidant properties of active substances contained in plant extracts can contribute to inhibiting or neutralizing free radical reactions [36,37,38].

**Figure 3:**
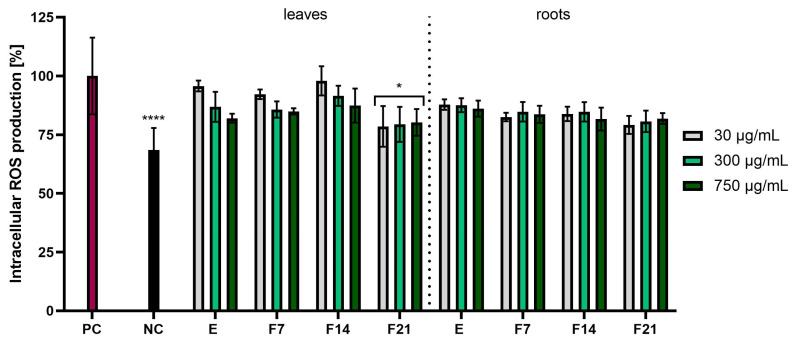
The effect of *R. sativus* L. leaf and root extracts (E) and ferments (F7, F14, and F21) at the concentrations of 30, 300, and 750 µg/mL on the intracellular level of reactive oxygen species (ROS) in fibroblasts (BJ). Data are presented as mean ± SD from three independent experiments, with each sample tested in triplicate. **** *p* < 0.0001, * *p* < 0.05 versus the PC (positive control).

**Figure 4:**
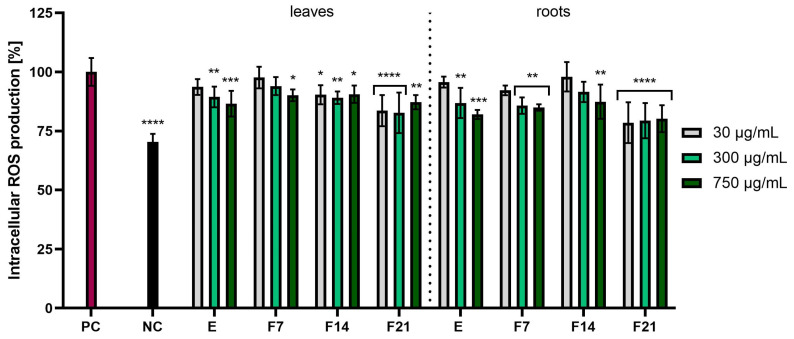
The effect of *R. sativus* L. leaf and root extracts (E) and ferments (F7, F14, and F21) at the concentrations of 30, 300, and 750 µg/mL on the intracellular level of reactive oxygen species (ROS) in keratinocytes (HaCaT). Data are presented as mean ± SD from three independent experiments, with each sample tested in triplicate. **** *p* < 0.0001, *** *p* < 0.001, ** *p* < 0.01, * *p* < 0.05 versus the PC (positive control).

The study assessed the possibility of reducing the intracellular level of reactive oxygen species in skin cells treated with radish extracts and ferments. These analyses were performed using the fluorogenic dye H_2_DCFDA, which in the presence of reactive oxygen species was oxidized and transformed into the highly fluorescent 2′,7′-dichlorofluorescein (DCF). This method was used to detect the total ROS pool because fluorescence activation occurs independently of the type of oxygen free radical. The analyses were performed on two cell lines: keratinocytes (HaCaT) and fibroblasts (BJ). Results were expressed as the percentage inhibition of ROS products relative to the positive control with H_2_O_2_ (PC) (100%). These studies confirmed the antioxidant properties, causing a reduction in reactive oxygen species in skin cells induced by H_2_O_2_. All tested radish extracts and ferments had the ability to reduce the level of reactive oxygen species. In the case of BJ cells, radish roots showed stronger antioxidant activity compared with radish leaves. Moreover, F21 showed the greatest potential for reducing the intracellular ROS level for leaves and roots, reaching 80.22 ± 5.70 and 81.87 ± 2.31%, respectively, at the concentration 750 µg/mL (Figure 3). In the case of HaCaT cells, ferments obtained from radish showed better antioxidant properties compared with extracts. The most favorable values were obtained for F21 compared with the positive control, leaves and roots, reaching 83.61 ± 6.58 and 78.51 ± 8.64%, respectively, at the concentration of 30 µg/mL (Figure 4). Moreover, the influence of fermentation time on reducing the ROS level was found. It was observed that in both BJ and HaCaT cells, the highest antioxidant potential was observed in F21 ferments for leaves and roots, while the lowest antioxidant potential was observed in extracts for leaves and roots (Figure 3 and Figure 4).

The antioxidant properties of *R. sativus* L. extract have been studied by other researchers. Lugasi et al. discovered that radish root extract had an antioxidant effect in vitro [39]. It was also suggested that the L-tryptophan enzyme isolated from raw radish extract may have an antioxidant effect [40]. Chromatographic analyses carried out on radish extracts and ferments showed the presence of compounds such as gallic acid, catechin, and rutoside (Table 1 and Table 2). Moreover, glucosinolates constitute an important class of secondary plant products with antioxidant properties [41]. They have proven antioxidant properties, which suggests their potential to lower the level of reactive oxygen species and reduce oxidative stress in cells. These results suggest that radish extracts contain a variety of antioxidant compounds that may potentially contribute to maintaining physiological balance in the body [42,43,44].

### 2.3. Cytotoxicity Assessment

In the next stage of the research, the cytotoxic properties of extracts and ferments obtained from the leaves and roots of *R. sativus* L. were assessed. The analyses were carried out on two cell lines—fibroblasts (BJ) and keratinocytes (HaCaT). The research was carried out using tests (Alamar Blue and Neutral Red). The performed analyses allowed the assessment of the cytotoxicity of the tested samples depending on the concentration and fermentation time. The first Alamar Blue (AB) test assessed the ability of living cells to convert resazurin into the reductive fluorescent resorufin, while the second Neutral Red (NR) test allowed for the detection of live cells by assessing the uptake of a red dye that stains lysosomes in living cells. The Supplementary Material also shows microscopic images of fibroblasts (Figure S4) i keratinocytes (Figure S5) taken using an inverted fluorescence microscope. 

**Figure 5:**
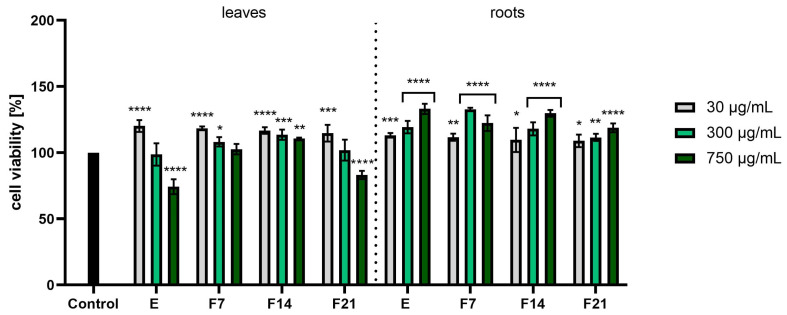
Effect of the increase in the concentrations of extracts (E) and ferments (F7, F14, and F21) from the leaves and roots of *R. sativus* L. (30–750 μg/mL) on cell viability (Alamar Blue assay) by cultured fibroblasts. Data are the mean ± SD of three independent experiments, each consisting of three replicates per test group. **** *p* < 0.0001, *** *p* < 0.001, ** *p* < 0.01, * *p* < 0.05 versus the control.

**Figure 6:**
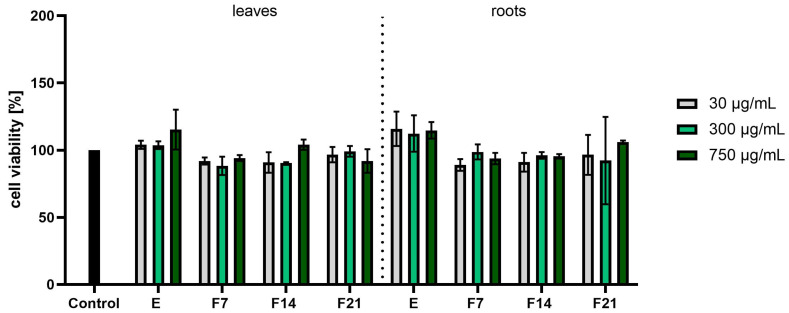
Effect of the increase in the concentrations of extracts (E) and ferments (F7, F14, and F21) from the leaves and roots of *R. sativus* L. (30–750 μg/mL) on cell viability (Alamar Blue assay) by cultured keratinocytes. Data are the mean ± SD of three independent experiments, each consisting of three replicates per test group.

When assessing the results of the Alamar Blue test (Figure 5), it was noticed that the roots had better proliferative properties than the leaves. The values were calculated as a percentage of the control (100%). At the higher concentrations tested, the viability of the fibroblasts was higher, reaching even 133.12 ± 3.86, 132.72 ± 1.33, and 129.76 ± 2.60% for E, F7, and F14, respectively, at the concentration of 750 µg/mL. In the case of leaves, higher concentrations resulted in lower cell viability values. Analyzing the cytotoxicity results for HaCaTs using the AB test (Figure 6), it was noticed that the extracts showed higher proliferation values at the highest tested concentrations, obtaining 115.28 ± 14.82 and 114.73 ± 6.13%, respectively. However, these results do not show statistical significance.

**Figure 7:**
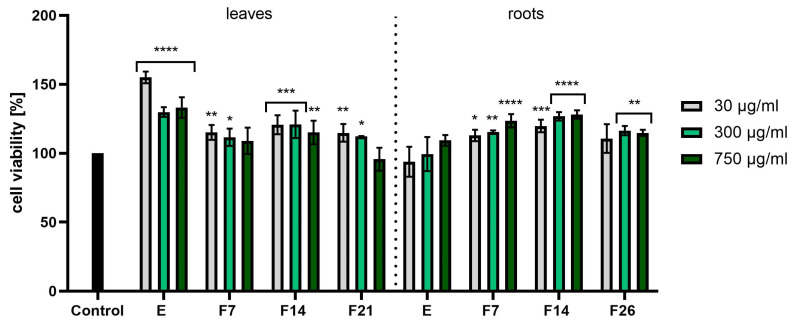
Effect of the increase in the concentrations of extracts (E) and ferments (F7, F14, and F21) from the leaves and roots of *R. sativus* L. (30–750 μg/mL) on cell viability (Neutral Red assay) by cultured fibroblasts. Data are the mean ± SD of three independent experiments, each consisting of three replicates per test group. **** *p* < 0.0001, *** *p* < 0.001, ** *p* < 0.01, * *p* < 0.05 versus the control.

**Figure 8:**
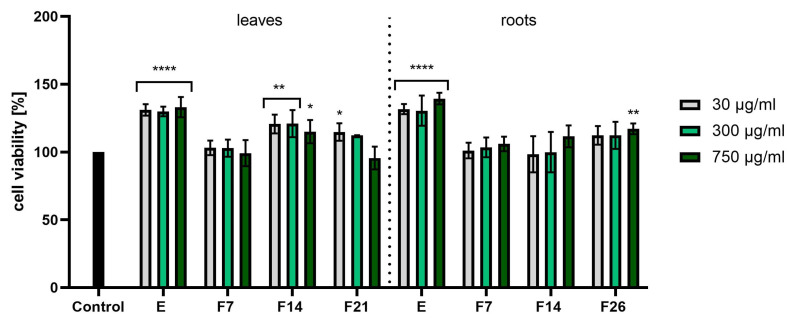
Effect of the increase in the concentrations of extracts (E) and ferments (F7, F14, and F21) from the leaves and roots of *R. sativus* L. (30–750 μg/mL) on cell viability (Neutral Red assay) by cultured keratinocytes. Data are the mean ± SD of three independent experiments, each consisting of three replicates per test group. **** *p* < 0.0001, ** *p* < 0.01, * *p* < 0.05 versus the control.

Figure 7 and Figure 8 have shown cytotoxicity results using the NR assay. In the case of fibroblast cells (Figure 7), E and F14 from leaves were characterized by the highest cell proliferation values, obtaining statistically significant differences (compared with cells not treated with the tested compounds). In turn, F7 and F14 from roots showed the greatest effect on cell viability, reaching 128.10 ± 3.06 and 123.50 ± 4.82% at a concentration of 750 µg/mL, respectively. When examining the cytotoxicity of keratinocytes using the NR test, it was noticed that extracts from both leaves and roots were characterized by the best proliferative properties. The viability values were around 130% in all tested concentrations. In the case of leaves, statistically significant results were obtained for F14. Moreover, the analyses performed showed that all tested compounds did not show any toxic effects on fibroblasts and keratinocytes.

To sum up, a beneficial effect of the tested extracts and ferments on the proliferation and viability of skin cells (fibroblasts and keratinocytes) was demonstrated. This was due to the presence of biologically active compounds contained in *R. sativus* L., which may have a beneficial effect on our skin. Roh et al. showed in their study that rasathiol, an isolated compound from *R. sativus* L. extract, shows the potential to increase extracellular matrix (ECM) synthesis in cultured skin fibroblasts. It enhances the production of type 1 collagen, elastin, and fibronectin, while promoting fibroblast proliferation. In addition, rasathiol’s effects are associated with activation of intracellular ERK1/2 and p38 MAPK signaling pathways [22]. These findings suggest that ingredients found in radish may protect the skin, potentially through antioxidant mechanisms. The important ingredients here are polyphenols, glucosinolates, and vitamins, including vitamin C, which acts as a cofactor in the enzymatic reactions needed for collagen synthesis and can also influence the process of keratinocyte differentiation [4,14]. In addition, the antioxidant compounds contained in the raw material may help protect fibroblasts against damage caused by free radicals [45]. Additionally, it should be remembered that the fermentation process significantly increases the bioavailability of active compounds, which increases the effectiveness of the analyzed extracts [46].

### 2.4. Assessment of Anti-Inflammatory Activity

Inflammation is mediated by several factors and cell signaling molecules that are released from macrophages. When macrophages are stimulated by endotoxins such as lipopolysaccharides (LPS), they release various pro-inflammatory mediators and cytokines such as nitric oxide (NO), prostaglandin E2 (PGE2), tumor necrosis factor α (TNF-α), interleukin (IL)-1β, and IL-6. Cyclooxygenase-2 (COX-2) is the predominant enzyme induced by various stimuli, including inflammation, growth factors, and cytokines [47,48]. To investigate the anti-inflammatory activity of *R. sativus* L. leaf and root extracts and ferments, the levels of pro-inflammatory interleukin (IL-1β) and cyclooxygenase-2 (COX-2) were monitored in human fibroblast cells treated with the bacterial lipopolysaccharide. The results were expressed as the percentage of inhibition of the cytokines tested relative to the control sample (cells not treated with extracts but treated with LPS).

**Figure 9:**
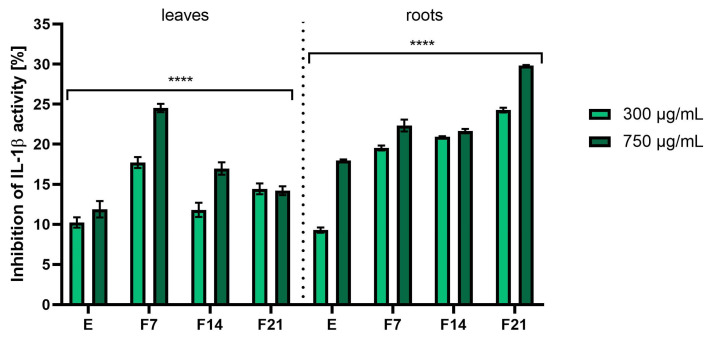
IL-1β inhibitory activity of *R. sativus* L. leaf and root extracts (E) and kombucha ferments (F7, F14, and F21) at concentrations of 300 and 750 µg/mL. Data are the mean of three independent experiments, each consisting of two replicates per treatment group. **** *p* < 0.0001 versus the control.

**Figure 10:**
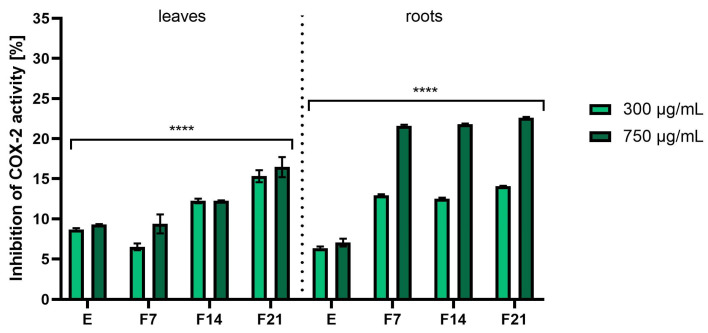
COX-2 inhibitory activity of *R. sativus* L. leaf and root extracts (E) and kombucha ferments (F7, F14, and F21) at concentrations of 300 and 750 µg/mL. Data are the mean of three independent experiments, each consisting of two replicates per treatment group. **** *p* < 0.0001 versus the control.

As shown in Figure 9, both extracts and ferments from radish leaves and roots have shown the ability to inhibit IL-1β. However, the roots were characterized by higher inhibition values. The highest values were recorded for F21 at a concentration of 750 µg/mL obtaining 29.82 ± 0.06%. In the case of leaves, 24.52 ± 0.53% of inhibition IL-1β activity was obtained for F7 at a concentration of 750 µg/mL. In addition, it was noted as the concentration of the tested compounds increased, the ability to inhibit IL-1β activity as well as COX-2 increased (Figure 10). In the case of the second tested cytokine, the results for the tested root extracts stood out significantly. The % of inhibition of COX-2 activity was 21.58 ± 0.16, 21.82 ± 0.05, and 22.62 ± 0.10% for F7, F14, and F21, respectively, at a concentration of 750 µg/mL. In the case of leaves, an increase in the inhibition value corresponded to the increase in fermentation time. The results also corresponded to the content of active compounds present in greater amounts in kombucha ferments (Table 1 and Table 2).

So far, radish has been subjected to many studies on the effects of cytokines involved in inflammatory processes in many cell lines. Jeon et al. showed that black radish extract significantly inhibited the increased production of NO, TNF-α, IL-6, and IL-1β by RAW 264.7 cells [49]. Moreover, the chloroform fraction of *R. sativus* L. leaves inhibited the expression of iNOS and COX-2 in RAW264.7 macrophages, thereby reducing inflammation [50]. Indologlucoside extracted from *R. sativus* L. seeds inhibited IL-6 production in TNF-α-stimulated MG-63 cells. Additionally, a phenylpropanoid isolated from radish seeds inhibited NO production by LPS-activated mouse BV-2 microglial cells [51,52]. Kook et al. demonstrated that *R. sativus* L. seeds had anti-inflammatory potential in LPS-stimulated macrophages and protected mice from septic injury. This study also shows that sinapic acid is one of the active substances responsible for the beneficial effect of radish seeds on the LPS-dependent inflammatory reaction [53]. Moreover, rasathiol isolated from *R. sativus* L., containing syringic acid, exhibits a number of biological activities, such as inhibition of COX-2 activity, antioxidant potential, and hepatoprotective effects [54]. The beneficial biological effects of radish extract are often attributed to phenolic compounds, glucosinolates, and flavonoids [18]. For example, flavonoids such as kaempferol and quercetin lead to a reduction in IL-6 release by macrophages. It has also been shown to have a positive effect on reducing the levels of TNF-α and COX-2, as well as increasing the secretion of IL-10 [55,56]. Kaempferol, present in extracts and ferments, inhibited the proliferation of both unstimulated and stimulated IL-1β-RASF, as well as IL-1β-induced mRNA and protein expression of MMP-1, MMP-3, COX-2, and PGE2. Kaempferol also inhibited ERK-1/2, p38, and JNK phosphorylation, as well as IL-1β-induced NF-κB activation. These results indicate that kaempferol inhibits the proliferation of synovial fibroblasts and the production of MMPs, COX-2, and PGE2, which are involved in inflammation and joint destruction in rheumatoid arthritis [57]. Catechins present in kombucha ferments are bioflavonoids and inhibit the expression of the IL-8 gene in respiratory epithelial cells. EGCG and other catechins can also inhibit proteins involved in inflammation, including TNF-α and xanthine oxidase [58]. Tumor necrosis factor (TNF)-induced fibroblast damage can also be alleviated with several polyphenols. Alpinumisoflavone (AIF), (-)-catechin, epigallocatechin-3-gallate (EGCG), and 7,8-dihydroxyflavone (7,8-DHF) inhibited TNF-induced MMP-1 synthesis and enhanced procollagen I. AIF and (-)-catechin inhibited the activity of NF-B and COX-2 [59,60]. The implication that extracts and kombucha ferments from *R. sativus* L. can impact the generation of the aforementioned pro-inflammatory cytokines suggests that this plant could serve as a component in cosmetics aimed at restraining or moderating inflammatory processes that often create challenges in the treatment of a variety of skin conditions.

### 2.5. Assessment of Antibacterial Activity

Due to the fact that bacterial infections play an important role in the development of many skin diseases, it is extremely desirable to assess the possibility of inhibiting the multiplication of various pathogenic organisms by raw materials used in the production of cosmetics. In addition to commonly used preservatives, there is an increasing search for natural compounds that could act as a natural preservative and prevent the growth of microorganisms in cosmetic preparations. Modern consumers are looking for natural cosmetics that do not contain preservatives. Therefore, cosmetics manufacturers are trying to develop preparations without the addition of preservatives or to create self-preserving cosmetics in which a selected raw material of plant origin serves as a preservative [61].

Radish is a plant whose antibacterial properties of leaves, seeds, and roots have been indicated by various authors [62,63,64,65,66,67]. However, there are no studies assessing the impact of both radish extracts and ferments on the multiplication of bacterial strains responsible for the occurrence of bacterial skin infections. Therefore, as part of this work, analyses of the antibacterial properties of the water extract and ferments from *R. sativus* L. (after 7, 14, and 21 days of fermentation) were carried out against strains whose excessive multiplication often leads to skin diseases.

The performed analyses indicated differences in the antibacterial activity of the tested extracts and ferments. In the case of both radish leaves and roots, stronger inhibition of the growth of the tested strains was observed for ferments, for which lower minimum inhibitory concentrations (MIC) were recorded compared with extracts. In the case of *R. sativus* L. root, both the extract and ferments inhibited the growth of *S. aureus*, *M. luteus*, *Y. enterocolitica*, and *P. aeruginosa*. Additionally, higher concentrations of F14 and F21 ferments also inhibited the growth of *S. capitis*. In the case of leaf ferments, the possibility of growth inhibition was observed for all tested bacterial strains. In the case of *R. sativus* L. leaf extract, no inhibition of multiplication was observed, only in the case of *S. capitis*. The obtained results also indicated that in the case of the tested ferments, the MIC values in most strains were lower compared with extracts (both from leaves and roots), which indicated their stronger antibacterial properties (Table 3 and Table 4). In the case of most of the tested strains, it was also shown that extending the fermentation time may increase the antibacterial potential of the tested samples, which was probably the result of the formation of various phytochemicals with antibacterial activity.

The ability of *R. sativus* L. to inhibit the growth of various microorganisms has also been confirmed by other authors. Rathour et al. indicated the antimicrobial effect of chloroform extract from *R. sativus* L. leaves on Salmonella enteritidis, Pseudomonas aeruginosa, and *Bacillus subtilis*. These authors also pointed out the stronger properties of chloroform extracts compared with petroleum ether or water extracts [62]. da Silva et al. pointed out the antibacterial properties of *R. sativus* L. leaf extract against Gram-positive bacteria such as *Enterococcus faecalis*, *Micrococcus luteus*, *Bacillus subtilis*, *Staphylococcus aureus*, *Bacillus cereus*, and *E. faecalis*. However, this research team did not observe any growth inhibition in the case of Gram-negative bacteria such as *Escherichia coli*, *Enterobacter cloacae*, *Proteus mirabilis*, or *Salmonella tiphy*. Additionally, analyses performed on fungi such as *Candida albicans* and *Saccharomyces cerevisiae* and the mycobacteria *Mycobacterium tuberculosis* and *Mycobacterium bovis* also did not indicate any inhibition of the multiplication of these microorganisms after the use of the tested extract. The lack of inhibition of the growth of Gram-negative bacteria may have been the result of differences in their structure compared with Gram-positive cells and the presence of an additional membrane in their structure tightly adhering to the peptidoglycan layer [63]. Jadoun et al. indicated the antibacterial properties of ethanolic extracts of *R. sativus* L. seeds against bacteria such as Streptococcus pyogenes, *Staphylococcus aureus*, *Escherichia coli*, Salmonella typhimurium, and Klebsiella pneumoniae. These authors attribute this activity mainly to sulfur-containing compounds contained in these seeds [64]. The possibility of inhibiting the growth of eight bacterial strains by radish seed extracts obtained by extraction with various solvents was also indicated by Ahmad et al. These authors pointed out the strongest antibacterial properties of ethanol and methanol extracts (compared with chloroform, ethyl acetate, water, and benzene extracts), which inhibited the growth *S. aureus*, *E. coli*, *P. aeruginosa*, *S. sonnei*, *S. typhi*, *P. vulgaris*, *K. pneumonie*, and *S. paratyphi*. The strongest inhibition of the growth of these microorganisms by the highest antibacterial effect of methanol and ethanol extracts may have been the result of a larger amount of biologically active compounds with antibacterial activity, such as phenolic compounds, tannins, saponins, flavonoids, and other secondary metabolites [65,66]. Duy et al. indicated the possibility of the aqueous extract to inhibit the growth of four species of fungi such as *Aspergillus flavus*, *Aspergillus niger*, *Aspergillus clavatus*, and *Fusarium solani* [26]. Sevindik et al. indicated different antimicrobial activity of extracts obtained from the aerial parts and roots of radish. Both types of extracts showed inhibitory effects against bacteria such as *S. aureus*, *E. faecalis*, *E. coli*, *P. aeruginosa*, and *A. baumannii*. Inhibition of the growth of fungal strains such as *C. albicans*, *C. krusei*, and *C. glabrata* was also observed. The antimicrobial effect observed by these authors was stronger for the root extract, for which lower minimum inhibitory concentration (MIC) values were obtained for all tested strains of microorganisms [67].

The mechanism of antibacterial action of plant extracts, including those obtained from *R. sativus* L., or individual phytochemicals may be multidirectional. It may include interactions with the cell membrane of microorganisms by increasing their permeability and disrupting cell metabolism, as well as inhibiting the uptake and absorption of nutrients necessary for the proper multiplication and growth of microorganisms [68,69,70]. Damage to the cell membrane of microorganisms may lead to changes in its permeability and thus impairment of various functions in bacterial cells. This may result in changes in electron transport, nucleic acid synthesis, and the activity of various bacterial enzymes. Moreover, some plant compounds can reach the cytoplasm and enter into chemical reactions with cellular components [71,72]. It should be noted, however, that the antibacterial activity of plant extracts or essential oils in cosmetic preparations may be slightly weaker than the in vitro activity of these raw materials. This is the result of possible interactions between them and individual cosmetic ingredients. These phytochemicals, due to their high affinity for the ingredients of cosmetic emulsions, are less available in the water–oil phase, which weakens the antibacterial effect [73].

Although the antibacterial activity of extracts from various parts of radish has already been proven by various authors, this work showed for the first time antibacterial activity against bacterial strains such as *Y. enterocolitica* and *S. capitis*, which often accompany skin diseases. Additionally, the study showed that fermenting extracts from *R. sativus* L. roots and leaves using kombucha may contribute to increasing their antibacterial activity.

### 2.6. Transepidermal Water Loss (TEWL) and Skin Hydration Measurements

The skin plays a key role in protecting the body from dehydration and environmental factors such as temperature fluctuations, humidity, and exposure to sunlight. UVB radiation may disturb the structure of the epidermis by thickening the stratum corneum, which leads to an imbalance in its permeability and an increase in transepidermal water loss (TEWL). Skin hydration is a key indicator of skin barrier function in both cosmetic formulations and skin diseases. Skin aging is often accompanied by decreased hydration, with hyaluronic acid (HA), an essential component of the extracellular matrix, playing a key role. Various factors, including hyaluronic acid and elastic fibres, influence skin hydration and elasticity [74,75].

**Figure 11:**
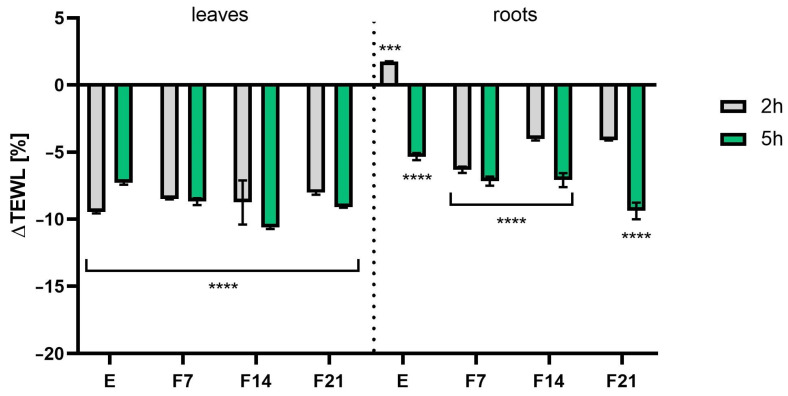
The influence of *R. sativus* L. leaf and root extracts (E) and ferments (F7, F14, and F21) on transepidermal water loss (TEWL). Data are the mean ± SD of three independent measurements. **** *p* < 0.0001, *** *p* = 0.0004 compared with the control field.

**Figure 12:**
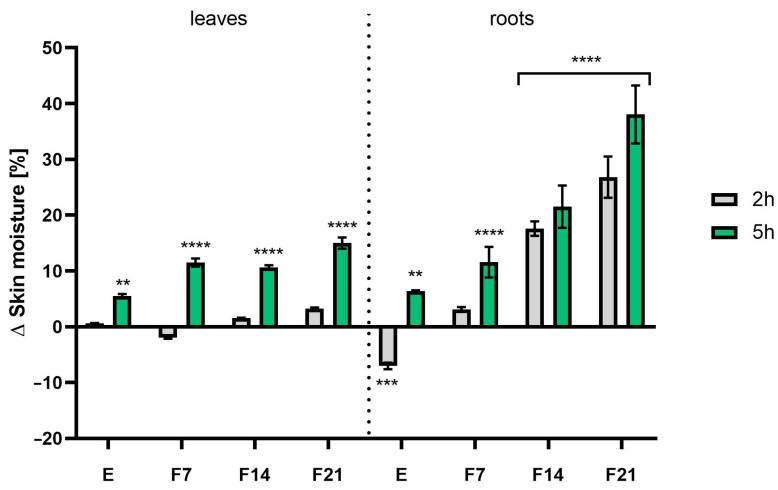
The influence of *R. sativus* L. leaf and root extracts (E) and ferments (F7, F14, F21) on transepidermal water loss (TEWL). Data are the mean ± SD of three independent measurements. **** *p* < 0.0001, *** *p* = 0.0006, ** *p* < 0.01 compared with the control field.

The study evaluated the effects of radish leaf and root extracts and ferments on basic skin parameters such as hydration and TEWL. Analyses were carried out at two time intervals, 2 h and 5 h after product application. The obtained extracts and ferments were tested at a concentration of 30 mg/mL. The results were expressed as a percentage of the control field (without any tested samples). The results showed that both extracts and ferments increased skin moisture and had a significant effect on skin barrier function by decreasing TEWL values. Comparing the measurement time, more favorable results were obtained 5 h after application, obtaining statistically significant differences. Moreover, radish roots showed better skin moisturizing properties both 2 and 5 h after application. What is more, the influence of fermentation time on the moisturizing ability was noted. The most favorable values were obtained for F21, obtaining 38.05 ± 5.18 and 26.82 ± 3.68% compared with the control for roots and leaves, respectively, 5 h after application (Figure 11). A similar relationship was observed when examining the level of transepidermal water escape from the epidermis. After 5 h after application of the preparation, statistically significant results were obtained for both the leaves and the roots, with more favorable TEWL values for kombucha ferments. As the fermentation time increased, the difference in TEWL levels compared with the control also increased, reaching 9.40 ± 0.62 and 10.63 ± 0.11% for F21 and F14 (roots and leaves), respectively (Figure 12).

In addition to the already known preservative properties of radish root extract, it is worth mentioning the cosmetic ingredient available under the INCI name *Leuconostoc/Radish root ferment lysate filtrate*, which is a lysate filtrate of a product obtained by fermentation of *R. sativus* L. roots by the microorganism Leuconostoc, which has conditioning properties on skin and hair. Studies have shown that secondary plant metabolites present in herbal extracts have a positive effect on skin barrier function [76]. As shown in Table 1 and Table 2, mainly phenolic acids and flavonoids present in the studied extracts and ferments can contribute to increased skin hydration [77,78,79]. Moreover, research indicates that fermentation products derived from SCOBY are rich in simple sugars containing hydroxyl groups in their structure. Thanks to this, these compounds act as valuable humectants with increased penetrating abilities because they are able to effectively reach deep into the epidermis compared with more complex substances that act primarily on the skin surface [80]. It appears that including kombucha in cosmetic recipes may help maintain optimal physiological skin condition and alleviate changes associated with dryness. Given that water loss can adversely affect the appearance of the skin and contribute to skin diseases, keeping the skin moisturized is important.

## 3. Materials and Methods

### 3.1. Plant Material and Fermentation Procedure

The roots and leaves of *R. sativus* L. were gathered from controlled and organic plantations where no chemical fertilizers or pesticides were utilized in cultivation. The starter culture of the kombucha tea fungus (SCOBY) was obtained from a commercial supplier in Poland. A symbiotic culture consisting of bacteria (*Acetobacter* and *Gluconobacter*) and yeasts (*Saccharomyces*, *Saccharomycodes*, *Schizosaccharomyces*, *Zygosaccharomyces*, *Dekkera*, *Candida*, *Torulospora*, *Kolectera*, *Pichia*, *Mycotorula*, and *Mycoderma*). Initially, sterile beakers were used to prepare the extracts by mixing 21 g of fresh roots and leaves with 700 mL of purified water at room temperature. To obtain the extracts, ultrasound-assisted extraction (UAE) was used according to the method described by Yang et al. [81], which consisted of placing the mixture in an ultrasonic bath (Digital Ultrasonic Cleaner, Berlin, Germany) for 30 min. The extracts were filtered through membrane filters into sterile glass beakers (1000 mL, height 18 cm, diameter 8 cm). Each beaker contained 200 mL of extract for fermentation. Sucrose at a final concentration of 10.0% *m*/*v* and kombucha tea fungi at a final concentration of 10% were then introduced into the beakers with the extracts. Fermentation was carried out for 7, 14, and 21 days, respectively, in separate beakers, at room temperature (approximately 25 °C). Ferments collected after 7, 14, and 21 days were labelled as F7, F14, and F21, respectively. The remaining unfermented extract was designated E.

### 3.2. Determination of Biologically Active Compounds

The primary metabolites were identified utilizing an ultra-high-performance liquid chromatography (UHPLC) system from the Infinity Series II, coupled with a DAD detector and an Agilent 6224 ESI/TOF mass detector (Agilent Technologies, Santa Clara, CA, USA). The HPLC parameters were configured as follows: A Titan RP18 reversed-phase column (Supelco, Sigma-Aldrich, Burlington, MA, USA) with dimensions of 10 cm length, 2.1 mm inner diameter, and a particle size of 1.9 µm was employed. The thermostat temperature was maintained at 30 °C, while the flow rate was set at 0.2 mL/min. The mobile phase comprised a mixture of water containing 0.05% formic acid (solvent A) and acetonitrile with 0.05% formic acid (solvent B). Separation of compounds was achieved through gradient elution, following this program: 0–9 min with 98% A transitioning to 95% A (and from 2% to 5% B), 9–24 min with 95% A transitioning to 92% A (and from 5% to 8% B), 24–45 min with 92% A transitioning to 85% A (and from 8% to 15% B), and 45–60 min with 85% A transitioning to 70% A (and from 15% B to 30% B). Chromatograms were recorded within the wavelength range of 200 to 600 nm. For LC–MS analysis, the ion source parameters were set as follows: drying gas temperature at 325 °C, drying gas flow rate at 8 L/min, nebulizer pressure at 30 psi, capillary voltage at 3500 V, fragmentator voltage at 220 V, and skimmer voltage at 65 V. Ion acquisition occurred within the range of 100 to 1050 *m*/*z*.

### 3.3. Assessment of Antioxidant Activity

#### 3.3.1. DPPH Radical Scavenging Assay

The antioxidant properties of the examined extracts and ferments derived from *R. sativus* L. leaves and roots were evaluated using the DPPH radical (1,1-diphenyl-2-picrylhydrazyl) assay, following a previously described method with modifications [82]. In brief, a 4 mM methanolic DPPH solution was combined with test samples at concentrations of 750, 1500, and 3000 µg/mL. The absorbance was measured at a wavelength of λ = 517 nm using a UV-VIS spectrophotometer (Thermo Fisher Scientific, Waltham, MA, USA). A control sample consisting of water with a DPPH solution was also included. Three independent experiments, each with triplicate measurements for every concentration, were conducted. The results are presented as a percentage of DPPH scavenging relative to the control.

#### 3.3.2. ABTS Scavenging Assay

The antioxidant properties of the examined extracts and ferments derived from *R. sativus* L. leaves and roots were also evaluated using a solution of 2,2’-azinobis-(3-ethylbenzothiazoline-6-sulfonic acid) (ABTS^+^, Sigma-Aldrich, Burlington, MA, USA), as described by a previous method with modifications [83]. Initially, a mixture of 7 mM ABTS^+^ solution and 2.4 mM potassium persulfate solution were prepared and incubated for 16 h in the dark at room temperature. This solution was then diluted in methanol to achieve an absorbance of approximately 1.0 at λ = 734 nm. Subsequently, the test samples at concentrations of 750, 1500, and 3000 µg/mL were combined with the ABTS^+^ solution, and the absorbance at λ = 734 nm was measured using a UV/VIS spectrophotometer (Thermo Fisher Scientific, Waltham, MA, USA). A control sample comprising methanol mixed with ABTS^+^ was included. Three independent experiments, with each concentration tested in triplicate, were conducted. The results are presented as a percentage of ABTS scavenging relative to the control.

#### 3.3.3. Detection of Intracellular Levels of Reactive Oxygen Species (ROS)

To evaluate the ability of *R. sativus* L. extracts and ferments to reduce the intracellular production of reactive oxygen species in keratinocyte and fibroblast cells, a fluorogenic 2′,7′-dichlorodihydrofluorescein diacetate (H_2_DCFDA) (Sigma-Aldrich, Burlington, MA, USA) dye was used. HaCaT and BJ cells were treated with analyzed samples dissolved in DMEM (Dulbecco’s Modified Eagle Medium, Biological Industries, Cromwell, CO, USA) at concentrations of 30, 300, and 750 µg/mL and incubated for 24 h. Following this incubation period, the samples were replaced with 10 µM H_2_DCFDA in serum-free DMEM medium in each well. Next, 5 mM hydrogen peroxide (H_2_O_2_) solution (resulting in a final concentration of 500 µM) dissolved in DMEM without serum was immediately added to the tested samples and the positive control. After 60 min of incubation, measurements were taken at an excitation wavelength of λ = 485 nm and an emission wavelength of λ = 530 nm using a microplate reader (FilterMax F5, Thermo Fisher Scientific, Waltham, MA, USA). The experiments were performed in triplicate and repeated independently three times for each sample. The results are presented as a percentage of positive control [84].

### 3.4. Cytotoxicity Analysis

#### 3.4.1. Cell Culture

To evaluate the cytotoxicity, two normal human skin cell lines were employed: keratinocytes (HaCaT, obtained from CLS Cell Lines Service, Eppelheim, Germany) and fibroblasts (BJ, acquired from ATCC^®^CRL-2522™, American Type Culture Collection, Manassas, VA, USA). These cell lines were cultured in DMEM (Dulbecco’s Modified Eagle Medium, Biological Industries, Cromwell, CO, USA). The medium was supplemented with 10% (*v*/*v*) fetal bovine serum (FBS, Biological Industries, Beit-Haemek, Israel) and 1% (*v*/*v*) antibiotics (100 U/mL penicillin and 1000 μg/mL streptomycin, Thermo Fisher Scientific, Waltham, MA, USA). Once the cells reached the appropriate confluence, HaCaT and BJ cells were seeded into 96-well plates and allowed to adhere for 24 h. Following this incubation period, the medium was replaced with tested extracts and ferments at concentrations of 30, 300, and 750 µg/mL and incubated for an additional 24 h.

#### 3.4.2. Alamar Blue Assay

The Alamar Blue test was performed to assess the viability of the skin cells. Following a 24 h incubation period of the skin cells with the tested extracts and kombucha ferments, a resazurin solution (Merck KGaA, Darmstadt, Germany) was added to each well at a concentration of 60 μM. Untreated cells cultured in DMEM served as controls. The plates were subsequently incubated for 2 h, after which the fluorescence was measured at λ = 570 nm using a microplate reader (Thermo Fisher Scientific, Waltham, MA, USA). Each sample was subjected to a triplicate test [84].

#### 3.4.3. Neutral Red Assay

The second assessment involved the Neutral Red uptake test. Following a 24 h incubation period, a Neutral Red dye (Sigma-Aldrich, Burlington, MA, USA) dissolved in DMEM (Dulbecco’s Modified Eagle Medium, Biological Industries, Cromwell, CO, USA) was introduced to each well of the 96-well plate containing skin cells along with the extracts and kombucha ferments, and the mixture was then incubated for 2 h. Subsequently, the cells were rinsed with sterile PBS, and the destaining buffer (comprising ethanol/acetic acid/water at proportions of 50%/1%/49%) was added. Absorbance readings were taken at λ = 540 nm using a microplate reader (Thermo Fisher Scientific, Waltham, MA, USA). Cells that did not receive treatment with test compounds served as controls. Each sample underwent testing three times [85].

### 3.5. Assessment of Anti-Inflammatory Activity

To assess the potential of extracts and ferments to inhibit interleukin-1β (IL-1 β) and cyclooxygenase-2 (COX-2) activity, spectrophotometric analysis was conducted using the Human IL-1β ELISA and Human COX-2 ELISA kits (Elabscience Biotechnology Inc., Houston, TX, USA). Initially, skin cells were seeded in six-well plates and incubated for 24 h at 37 °C. Following this incubation period, the tested compounds dissolved in DMEM (at concentrations of 300 and 750 µg/mL) were added, and the plates were further incubated for 24 h. Concurrently, cells treated with the test compounds were exposed to bacterial lipopolysaccharide (LPS) from *Escherichia coli* O111:B4 at a concentration of 5 µg/mL for 24 h, while untreated extracts and ferments were also treated with LPS. Subsequently, the cells were lysed with RIPA buffer. The lysed cells were then subjected to a sandwich ELISA following the manufacturer’s protocol. The inhibition of IL-1 β and COX-2 activity was determined by comparing the protein levels in untreated cells with those after treatment with the tested samples. Protein concentrations were calculated based on the standard curves obtained. Duplicate samples were analyzed for each experiment.

### 3.6. Assessment of Antibacterial Activity

The antibacterial activity of extracts and ferments from *R. sativus* L. leaves and roots was assessed by determining the minimum inhibitory concentration (MIC) for the growth of selected bacterial strains. The analyzed strains, which were obtained from the American Type Culture Collection (Manassas, VA, USA), were *Pseudomonas aeruginosa* ATCC^®^ 35032, *Staphylococcus aureus* ATCC BAA-2312, *Micrococcus luteus* ATCC^®^ 10240™, *Staphylococcus capitis* ATCC^®^ 146™, *Staphylococcus epidermidis* ATCC^®^ 49134™, *Corynebacterium xerosis* ATCC^®^ 373, and *Yersinia enterocolitica* ATCC 27729. To assess the antibacterial properties, the broth microdilution technique with a growth indicator p-iodonitrotetrazolium violet (INT, Merck KGaA, Darmstadt, Germany) was used. The analysis was performed based on the procedure described by Eloff [86]. Briefly, the tested samples (extracts and ferments F7, F14, and F21) were serially diluted with broth to obtain concentrations ranging from 25 to 3000 µg/mL. The prepared sample dilutions were placed in 96-well microplates. In the next step, a suspension of a specific bacterial strain containing 5 × 10^4^ colony-forming units was added to each well. As part of the analyses, a sterility control well, an antibiotic well, and a growth control well were also prepared for each microorganism. The plates were then incubated in an incubator at 37 °C for 24 h. After this time, 40 µL of INT solution with a concentration of 0.4 mg/mL was added to the wells. The prepared plates were placed back in the incubator for 30 min. The MIC value was the minimum concentration of extracts or ferments from radish leaves or roots that were able to inhibit the growth of the tested bacterial strains. As part of the analyses, three independent experiments were performed.

### 3.7. Transepidermal Water Loss (TEWL) and Skin Hydration Measurements

The measurements of transepidermal water loss (TEWL) and skin hydration were conducted using the TEWAmeter TM 300 probe and Corneometer CM825 probe, respectively, all connected to an MPA adapter (Courage + Khazaka Electronic, Köln, Germany). This study involved 10 volunteers. Areas measuring 2 × 2 cm were marked on the forearm skin of the volunteers. Subsequently, 0.2 mL of the extracts and kombucha ferments were applied to three fields. One field (control field) remained untreated with any sample. Measurements of hydration skin and TEWL were taken after 2 and 5 h. The final results were obtained by calculating the arithmetic mean from each volunteer, based on 5 independent measurements for skin hydration and 20 measurements for TEWL. The results are presented as % of control (field not treated with the tested compounds) [37].

### 3.8. Statistical Analysis

The data are presented as means ± SD of three independent experiments. The obtained experimental data were analyzed with one-way analysis of variance (ANOVA) followed by Dunnett’s post-test. The statistical significance was determined at **** *p* < 0.0001, *** *p* < 0.001, ** *p* < 0.01, and * *p* < 0.05 compared with the control. The statistical analysis was performed using GraphPadPrism 8.4.3. (GraphPad Software, Inc., San Diego, CA, USA).

## 4. Conclusions

In the cosmetic industry, radish extracts are known for their beneficial effects on the skin. Our research showed that fermented plant extracts offer many possibilities in addition to leaf and root extracts. Studies available in the literature suggest that substances other than black and green tea can serve as suitable substrates for kombucha fermentation. The results of this study indicated that leaf and root extracts and kombucha ferments showed increased levels of biologically active compounds compared with aqueous extracts alone. Both radish leaf and root extracts showed the presence of active compounds, in particular catechins, epicatechins, and gallocatechins. The results indicated that they may be valuable antioxidants, have anti-inflammatory properties, and help inhibit the growth of bacteria involved in inflammatory processes in the skin. In addition, the study showed positive effects on the skin, increasing skin hydration and lack of cytotoxicity toward keratinocytes and fibroblasts. Furthermore, the fermentation time was shown to affect both the content of active compounds and biological properties, with F21 almost always having the highest polyphenol content. The biological properties may classify the kombucha fermentation extract as a beneficial cosmetic raw material. Although the tests showed, among other things, no toxicity to skin cells and a positive effect on skin hydration, the tests had to be extended with an additional toxicological assessment aimed at assessing safety in cosmetic applications.

## Figures and Tables

**Table 1:** Quantitative analysis of *R. sativus* L. leaf extract and kombucha ferments performed using UHPLC/DAD/ESI-MS. The values represent means ± standard deviation (SD) of triplicate.

Rt (min)	Observed Ion Mass [M-H]-/(Fragments)	Δ ppm	Formula	Identified	Extract (µg/mL)	Ferment 7	Ferment 14	Ferment 21
1.49	195.05182	4.05	C_6_H_12_O_7_	Gluconic acid	-	+++	+++	+++
3.75	169.01443 (125)	1.08	C_7_H_6_O_5_	Gallic acid	-	0.94 ± 0.04 ^a^	1.44 ± 0.08 ^b^	2.05 ± 0.09 ^c^
6.17	315.07299 (153)	2.64	C_13_H_16_O_9_	Dihydroxybenzoic hexoside	+	+	+	+
7.68	305.06789	3.97	C_15_H_14_O_7_	Gallocatechin	-	+	+	+
12.72	305.06731	2.07	C_15_H_14_O_7_	Gallocatechin	-	24.92 ± 1.25 ^a^	24.28 ± 1.11 ^a^	23.95 ± 1.45 ^a^
13.28	289.07189	0.44	C_15_H_14_O_6_	Catechin	-	8.18 ± 0.44 ^a^	7.97 ± 0.39 ^a^	7.89 ± 0.31 ^a^
17.38	289.07256	2.75	C_15_H_14_O_6_	Epicatechin	-	5.9 ± 0.34 ^a^	7.74 ± 0.31 ^b^	8.11 ± 0.39 ^b^
17.68	295.04569 (179.133)	−0.85	C_13_H_12_O_8_	Caffeoylmalic acid	4.94 ± 0.25 ^a^	3.71 ± 0.14 ^b^	3.72 ± 0.21 ^b^	4.08 ± 0.20 ^b^
21.31	279.05119 (163.133)	0.58	C_13_H_12_O_7_	p-coumaroylmalic acid	6.53 ± 0.31 ^a^	4.46 ± 0.22 ^b^	4.58 ± 0.21 ^b^	5.11 ± 0.26 ^c^
24.16	309.06198 (193.133)	1.25	C_14_H_14_O_8_	Feruloylmalic acid	4.65 ± 0.25 ^a^	3.61 ± 0.18 ^b^	3.41 ± 0.17 ^b^	3.82 ± 0.20 ^b^
28.06	593.15188 (283.447)	1.15	C_27_H_30_O_15_	Kaempferol-3-O-glucoside-7-O-rhamnoside	4.91 ± 0.22 ^a^	4.93 ± 0.23 ^a^	4.91 ± 0.21 ^a^	5.15 ± 0.26 ^a^
29.17	609.14650	0.64	C_27_H_30_O_16_	Rutoside	+	1.54 ± 0.01 ^a^	2.07 ± 0.05 ^b^	2.12 ± 0.04 ^b^
30.32	593.15081 (283)	−0.65	C_27_H_30_O_15_	Kaempferol-3-rutoside	5.88 ± 0.31 ^a^	5.15 ± 0.22 ^b^	5.76 ± 0.23 ^a^	5.88 ± 0.25 ^a^
31.40	563.14124 (283)	1.08	C_26_H_28_O_14_	Kaempferol derivatives	4.03 ± 0.20 ^a^	3.17 ± 0.16 ^b^	3.92 ± 0.18 ^a^	3.98 ± 0.17 ^a^
36.71	577.15698 (284.431)	1.21	C_27_H_30_O_14_	Kaempferitrin	9.57 ± 0.51 ^a^	8.67 ± 0.48 ^a^	9.46 ± 0.41 ^a^	10.03 ± 0.55 ^a^
37.98	887.22917 (283)	4.53	C_41_H_44_O_22_	Kaempferol derivatives	+	+	+	+
55.22	327.21887	3.57	C_18_H_32_O_5_	Fatty acid deriv.	++	++	++	++
58.93	329.23440	3.19	C_18_H_34_O_5_	Fatty acid deriv.	++	++	++	++
64.79	331.24989	2.69	C_18_H_36_O_5_	Fatty acid deriv.	+++	++	++	++
65.86	265.14508	2.06	C_15_H_22_O_4_	Fatty acid deriv.	+++	++	++	++

“-” not detected; “+” detected; “++” detected in a medium amount; “+++” detected in high amount. The presence of different letters on the same lines indicates a statistically significant difference (*p* < 0.05).

**Table 2:** Quantitative analysis of *R. sativus* L. root extract and kombucha ferments performed using UHPLC/DAD/ESI-MS. The values represent means ± standard deviation (SD) of triplicate.

Rt (min)	Observed Ion Mass [M-H]-/(Fragments)	Δ ppm	Formula	Identified	Extract (µg/g)	Ferment 7	Ferment 14	Ferment 21
1.49	195.05170	3.44	C_6_H_12_O_7_	Gluconic acid	+	++	++	+++
3.81	169.01451 (125)	1.55	C_7_H_6_O_5_	Gallic acid	-	0.68 ± 0.11 ^a^	1.16 ± 0.07 ^b^	1.71 ± 0.03 ^c^
7.68	305.06795	4.16	C_15_H_14_O_7_	Gallocatechin	-	2.61 ± 0.16 ^a^	2.53 ± 0.17 ^a^	4.18 ± 0.09 ^b^
12.74	305.06748	2.63	C_15_H_14_O_7_	Gallocatechin	7.03 ± 0.10 ^a^	14.86 ± 0.45 ^b^	24.15 ± 1.38 ^c^	22.02 ± 0.41 ^c^
13.31	289.07204	0.96	C_15_H_14_O_6_	Catechin	-	1.46 ± 0.03 ^a^	1.76 ± 0.07 ^b^	3.41 ± 0.16 ^c^
17.38	289.07273	3.34	C_15_H_14_O_6_	Epicatechin	+	5.63 ± 0.06 ^a^	6.40 ± 0.12 ^b^	8.04 ± 0.12 ^c^
29.16	609.14663	0.85	C_27_H_30_O_16_	Rutoside	-	0.87 ± 0.01 ^a^	0.85 ± 0.04 ^a^	0.98 ± 0.03 ^b^
29.81	463.08835	0.32	C_21_H_20_O_12_	Quercetin glucoside		0.21 ± 0.01 ^a^	0.19 ± 0.01 ^a^	0.23 ± 0.01 ^b^
30.34	933.26412	−1.92	C_43_H_48_O_23_	Pelargonidin deriv.	+	+	+	+
35.57	1019.26477	−1.51	C_46_H_50_O_26_	Pelargonidin deriv.	++	++	++	++
40.89	1019.26610	−0.20	C_46_H_50_O_26_	Pelargonidin deriv.	++	+++	+++	+++
43.32	723.50602	1.03	C_41_H_72_O_10_	Unidentified	+++	+++	+++	+++
48.44	836.58678	0.38	C_44_H_85_O_14_	Unidentified	+++	+++	+++	+++
55.39	327.21887	3.57	C_18_H_32_O_5_	Fatty acid deriv.	+	+	+	+
58.93	329.23440	3.19	C_18_H_34_O_5_	Fatty acid deriv.	+	+	+	+
64.81	331.25007	3.23	C_18_H_36_O_5_	Fatty acid deriv.	+++	++	++	++
65.89	265.14508	2.06	C_15_H_22_O_4_	Fatty acid deriv.	+++	++	++	++

“-” not detected; “+” detected; “++” detected in a medium amount; “+++” detected in high amount. The presence of different letters on the same lines indicates a statistically significant difference (*p* < 0.05).

**Table 3:** Minimum inhibitory concentration (MIC) values of *R. sativus* L. root extract and ferments against the tested bacteria.

Test Microorganism	Minimum Inhibitory Concentrations (MIC) [µg/mL]			
	Extract	Ferment 7	Ferment 14	Ferment 21
*Staphylococcus aureus*	100	100	50	100
*Staphylococcus epidermidis*	nd	nd	nd	nd
*Staphylococcus capitis*	nd	nd	800	1000
*Micrococcus luteus*	400	100	100	200
*Corynebacterium xerosis*	nd	nd	nd	nd
*Yersinia enterocolitica*	200	200	50	50
*Pseudomonas aeruginosa*	50	100	50	50

nd—not detected.

**Table 4:** Minimum inhibitory concentration (MIC) values of *R. sativus* L. leaves extract and ferments against the tested bacteria.

Test Microorganism	Minimum Inhibitory Concentration (MIC) [µg/mL]			
	Extract	Ferment 7	Ferment 14	Ferment 21
*Staphylococcus aureus*	400	200	100	100
*Staphylococcus epidermidis*	400	600	400	400
*Staphylococcus capitis*	nd	nd	800	1000
*Micrococcus luteus*	50	50	50	50
*Corynebacterium xerosis*	600	400	400	200
*Yersinia enterocolitica*	100	50	50	50
*Pseudomonas aeruginosa*	600	600	400	400

nd—not detected.

## Data Availability

Data are contained within the article.

## Supplementary Materials

The following supporting information can be downloaded at https://www.mdpi.com/article/10.3390/ijms25115622/s1.
