# Supplementary material for: Comparison of Anti-Inflammatory and Antibacterial Properties of Raphanus sativus L. Leaf and Root Kombucha-Fermented Extracts
Source: Int J Mol Sci. 2024 May 22;25(11):5622. doi: 10.3390/ijms25115622 (PMC11171837; doi:10.3390/ijms25115622)
Supplement: Supplementary file 1 [file ijms-25-05622-s001.zip › ijms-3004679-supplementary.pdf]

Supplementary materials

Comparison of Anti-Inflammatory and Antibacterial Properties of *Raphanus sativus* L. Leaf and Root Kombucha-Fermented Extracts

Aleksandra Ziemlewska [1], Martyna Zagórska-Dziok [1], Agnieszka Mokrzyńska [1], Zofia Nizioł-Łukaszewska [1], Dariusz Szczepanek [2], Ireneusz Sowa [3] and Magdalena Wójciak [3,*]

[1] Department of Technology of Cosmetic and Pharmaceutical Products, Medical College, University of Information Technology and Management in Rzeszow, Sucharskiego 2, 35-225 Rzeszow, Poland; aziemlewska@wsiz.edu.pl (A.Z.); mzagorska@wsiz.edu.pl (M.Z.-D.); amokrzynska@wsiz.edu.pl (A.M.); zniziol@wsiz.edu.pl (Z.N.-Ł.)

[2] Department of Neurosurgery and Paediatric Neurosurgery, Medical University of Lublin, 20-090 Lublin, Poland; dariusz.szczepanek@umlub.pl

[3] Department of Analytical Chemistry, Medical University of Lublin, Aleje Raclawickie 1, 20-059 Lublin, Poland; i.sowa@umlub.pl

* Correspondence: magdalena.wojciak@umlub.pl

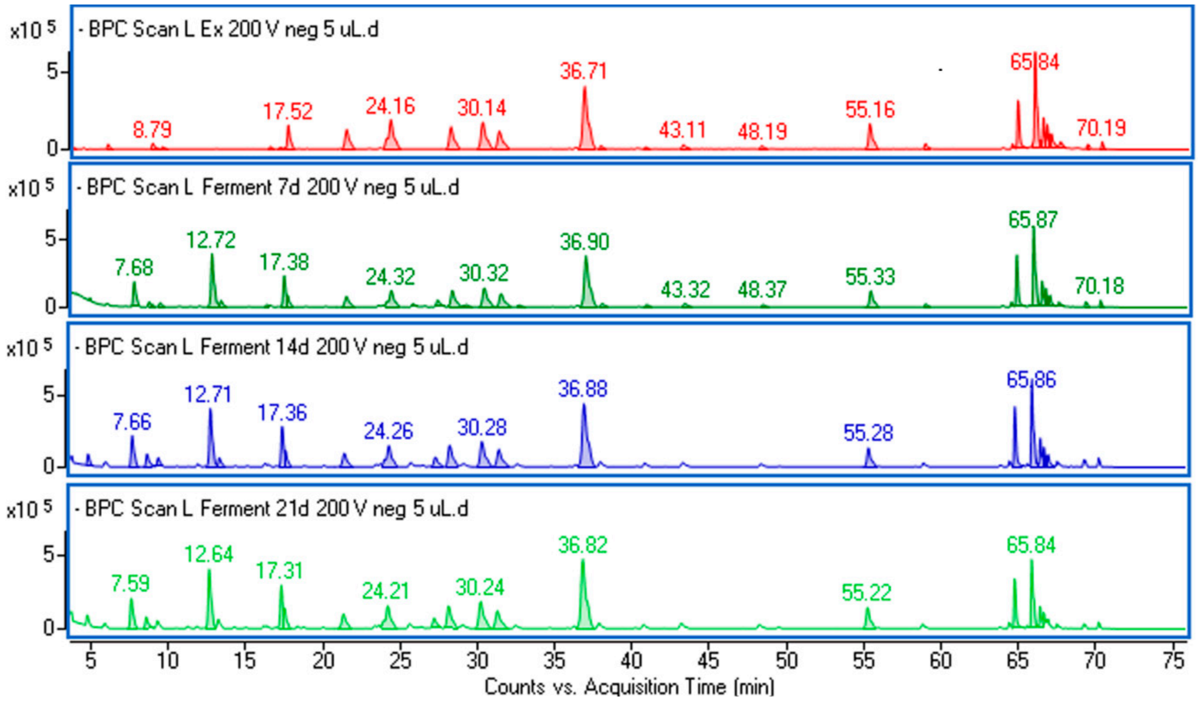

Figure S1. Example of chromatograms obtained for extract and fermented extract from *R. sativus* L. leaves

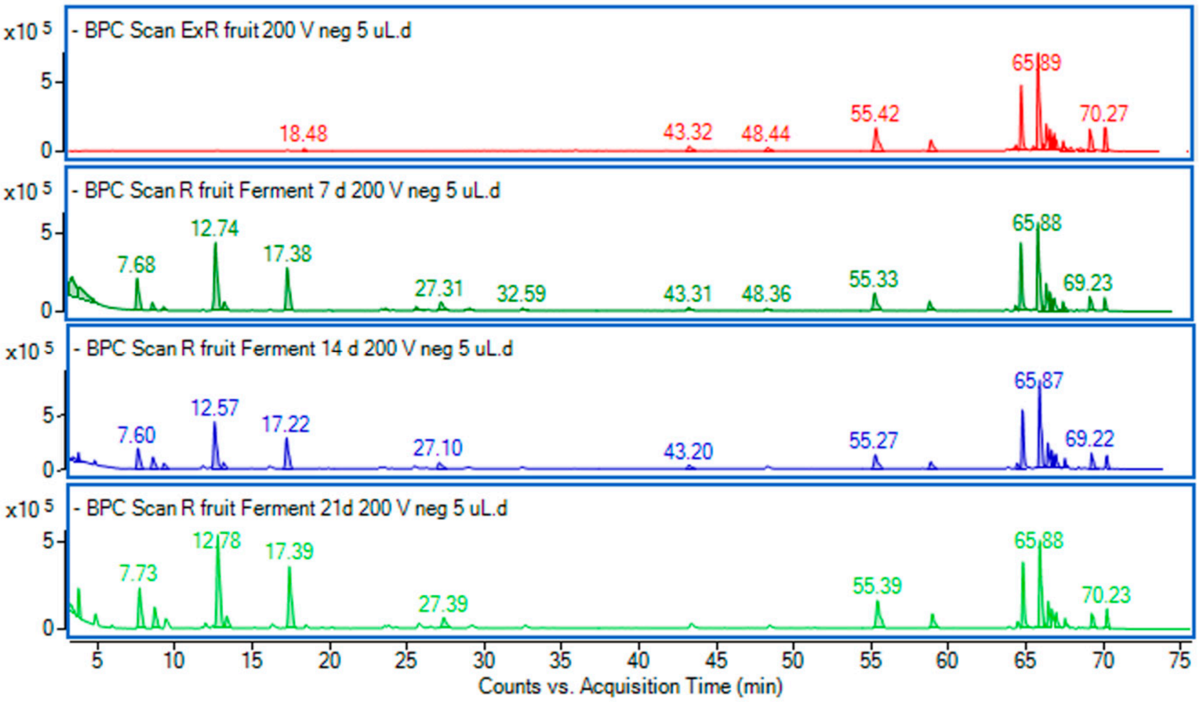

Figure S2. Example of chromatograms obtained for extract and fermented extract from *R. sativus* L. roots

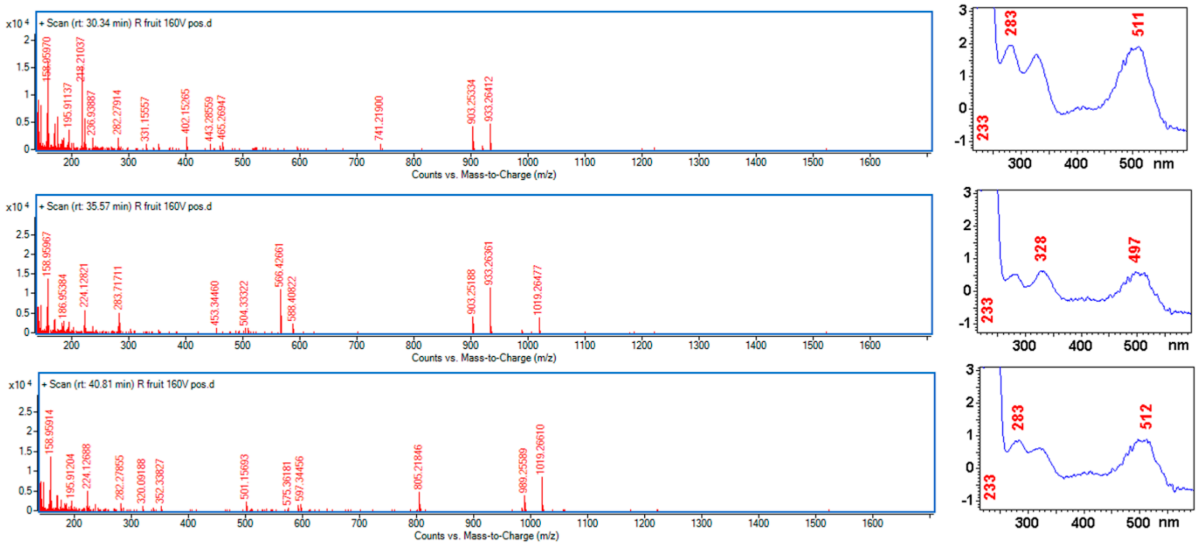

Figure S3. MS and UV-Vis spectra of anthocyanins found in the extract and fermented extract from *R. sativus* L. roots.

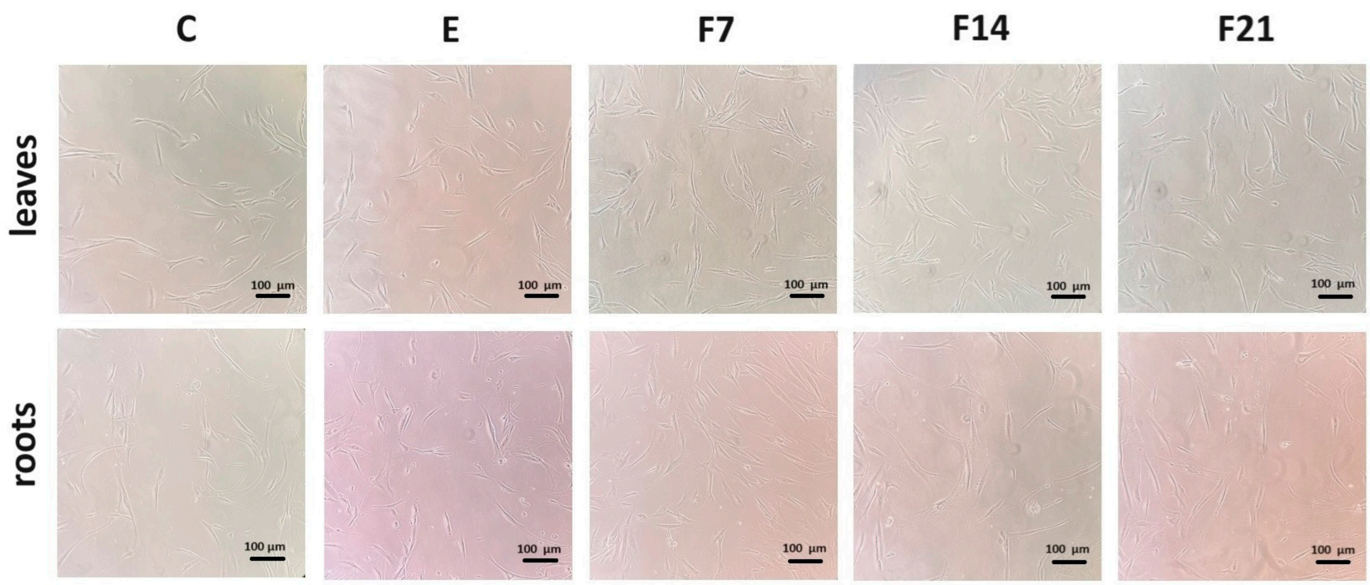

Figure S4. Microscopic images of fibroblasts (BJ) taken using an inverted fluorescence microscope. The images show the extract and ferment from the leaves and roots of *R. sativus* L.

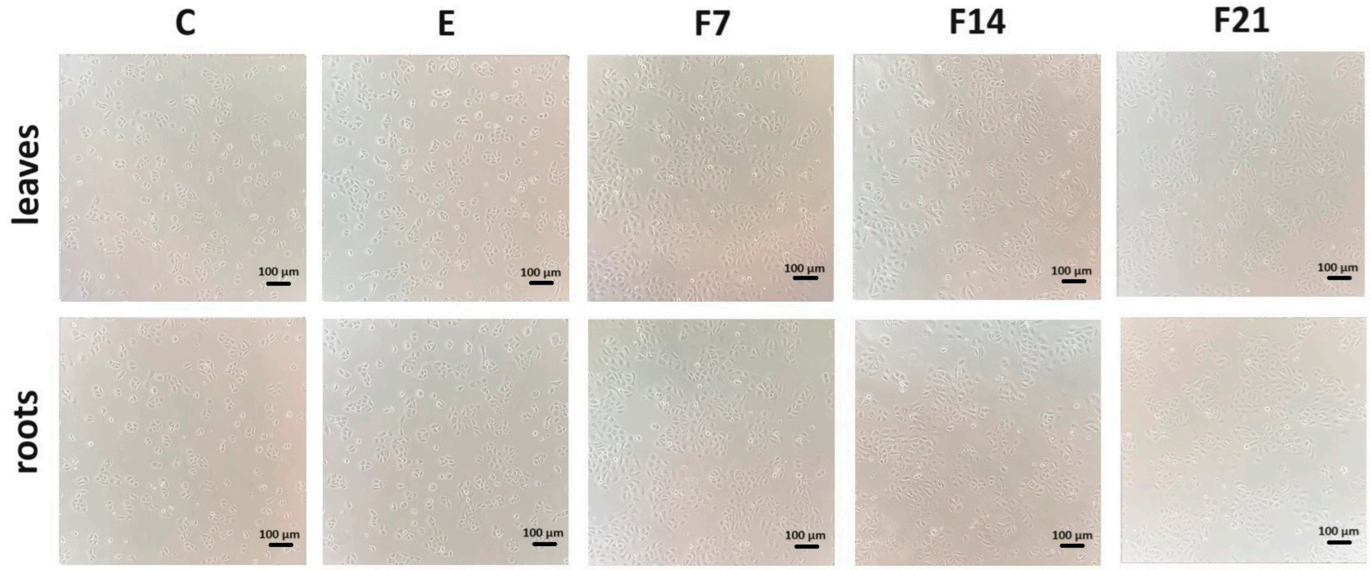

Figure S5. Microscopic images of keratinocytes (HaCaT) taken using an inverted fluorescence microscope. The images show the extract and ferment from the leaves and roots of *R. sativus* L.
